# Impact of OM-85 Given during Two Consecutive Years to Children with a History of Recurrent Respiratory Tract Infections: A Retrospective Study

**DOI:** 10.3390/ijerph16061065

**Published:** 2019-03-25

**Authors:** Susanna Esposito, Sonia Bianchini, Ilaria Polinori, Nicola Principi

**Affiliations:** 1 Pediatric Clinic, Department of Surgical and Biomedical Sciences, Università degli Studi di Perugia, 06132 Perugia, Italy; bianchini.sonia@fastwebnet.it (S.B.); ilaria.polinori@hotmail.it (I.P.); 2 Università degli Studi di Milano, 20146 Milan, Italy; nicola.principi@unimi.it

**Keywords:** bacterial lysate, OM-85, pediatric pulmonology, recurrent respiratory infections, respiratory tract infection

## Abstract

In otherwise healthy infants and young children, respiratory tract infections (RTIs) are extremely common. Clinical data have shown that OM-85 could prevent respiratory recurrences in children. However, further studies are needed to explore the true importance of OM-85 in clinical practice. In addition, an unsolved problem is the efficacy of OM-85 when it is administered for two consecutive years. Moreover, another open question is the safety of OM-85 when co-administered with the influenza vaccine. In order to solve these unanswered issues, 200 children aged three to six years with a history of recurrent RTIs, defined as at least six documented episodes of acute RTI in a single year, who had received OM-85 (Broncho-Vaxom^®^; OM Pharma, a Vifor Pharma Group Company, Geneva, Switzerland) for two consecutive years (3.5 mg once a day for 10 days for 3 months of each year) were selected and matched based on age, sex, and period of evaluation with children with recurrent RTIs who did not receive OM-85. In the group of children treated with OM-85, the number of patients who did not experience any new episode of RTI, as well as the number of RTIs, wheezing episodes, medical visits, and prescribed antibiotic courses, were significantly lower than that in the group not treated with OM-85. The results were similar in the first and second year of OM-85 administration. A minority of patients showed mild adverse events, and the safety profile was overall good, including in the 49 children who received the influenza vaccination within one month from the beginning of the first cycle of OM-85. Our data suggest that OM-85 can effectively and safely reduce the risk of new infective episodes in children with recurrent RTIs and that a second yearly course of lysate administration can be useful to maintain protection, particularly when the diagnosis of recurrent RTIs is made in younger children for whom it is likely that definitive maturation of the immune system still requires a long time.

## 1. Introduction

In otherwise healthy infants and young children, respiratory tract infections (RTIs) are extremely common. In a great number of cases, they are frequently recurrent, and despite being generally mild and spontaneously resolving in a few days, they can cause significant medical, social, and economic problems for the child, his or her family, and society [1]. Although genetic characteristics and several environmental factors (i.e., exposure to air pollutants, admission to day care centers, and lack of breastfeeding) play a role in favoring RTI development, immunological immaturity is the most important factor that explains the high incidence and tendency of these infections to recur. This conclusion is supported by the evidence that the incidence of RTIs tends to decrease with increasing age and becomes negligible during school age when the immune system is almost completely mature [2].

To reduce the total burden of RTIs, the prevention of recurrences through the elimination of environmental factors and systematic use of available vaccines is strongly suggested. Unfortunately, these measures are only partially effective [3]. To improve protection, boosting immune system activity has been suggested. OM-85, a lysate of 21 common bacterial respiratory pathogens, has been considered a possible measure in this regard [4,5,6]. In both in vitro and experimental animals, OM-85 activates mucosal dendritic cells in gastrointestinal Peyer’s patches, stimulating the activity of both innate and adaptive immune system responses against diverse pathogens, including influenza and respiratory syncytial virus, as well as the bacterial superinfection following influenza [4,5,6]. In general, animals administered OM-85 are protected from respiratory infections [7,8]. Moreover, clinical data have suggested that OM-85 could prevent respiratory recurrences in children. Several years ago, Schaad reported in a meta-analysis of eight randomized controlled trials, that children treated with OM-85 had significantly and consistently fewer cases of recurrent RTIs [9]. This finding was confirmed by a more recent meta-analysis of 53 randomized controlled trials involving 4851 pediatric patients [10]. However, it was concluded that although it was clearly evidenced that OM-85 was safe and well tolerated, further studies were needed to explore the true importance of OM-85 in clinical practice.

An unsolved problem is the efficacy of OM-85 when it is administered for consecutive years. Many children with RTIs receive OM-85 with benefit during a single winter season. Due to their young age, these children remain at risk of RTIs in the following year and should receive a second course of this immunomodulator to prevent recurrences. However, the results of a second OM-85 course are not defined. Moreover, unsolved is the problem of the safety of OM-85 when co-administered with the influenza vaccine [11]. This study was planned to solve these unanswered problems.

## 2. Materials and Methods

### 2.1. Study Design

This study was a retrospective controlled study performed in Italy to assess whether the efficacy of OM-85 (Broncho-Vaxom^®^; OM Pharma, a Vifor Pharma Group Company; Geneva, Switzerland) in reducing the number of new RTI episodes in children with a history of recurrent RTIs persists over time when it is administered in two consecutive years. The study was approved by the Ethics Committee of the Fondazione IRCCS Ca’ Granda Ospedale Maggiore Policlinico, Milan, Italy. The participants’ parents or legal guardians provided written informed consent before the children were enrolled.

### 2.2. Study Population

The study involved children aged 3 to 6 years with a history of recurrent RTIs, defined as at least 6 documented episodes of RTI (no more than one episode of community acquired pneumonia) in a single year. After the diagnosis, patients had to be regularly followed in the outpatient section for respiratory disorders at the university-affiliated pediatric center of Milan (Fondazione IRCCS ca’ Granda Ospedale Maggiore Policlinico) according to the standard protocol established for these patients. Practically, it was established that every time a child with recurrent RTIs experienced a febrile episode accompanied by symptoms suggesting RTI, parents had to contact the center for a visit or convey to the center by telephone the diagnosis and treatment prescribed by the primary care pediatricians who treated the child. Children with recurrent RTIs associated with malformations of the respiratory tract, chronic diseases of the lung, kidney and liver, primary or secondary immunodeficiencies, cancer, and malnutrition were excluded.

Patients fulfilling inclusion and exclusion criteria who had received OM-85 for two consecutive years (3.5 mg once a day for 10 days in September, October, and November of each year) during the period 2012–2017 were selected. Each child was matched based on age, sex, and period of evaluation, with a child with recurrent RTIs who did not receive OM-85. After selection of children who had received OM-85, matching was done with children with recurrent RTIs regularly followed in the same center. From the medical records of each child, influenza vaccination as well as incidence rates and characteristics (type of infection, need for medical visits, drug treatment) of RTIs diagnosed in children receiving prophylaxis and matched controls during the period from 1 September to 31 March in each of the two consecutive years were retrieved.

Efficacy of treatment was measured by comparing the total number of children with new RTI episodes, the number and type of RTIs diagnosed, and the number of children requiring antibiotics during the whole study period (from 1 October to 31 March) of each of the two consecutive years between patients and controls. Analysis also included the atopy status. Safety of OM-85 was assessed based on clinical manifestations that occurred during the periods of OM-85 administration and reported as potentially associated with the bacterial lysate.

### 2.3. Statistical Analysis

The continuous variables were expressed as the mean values ± standard deviation (SD) and were analyzed using a two-sided Student’s test if they were normally distributed (on the basis of the Shapiro–Wilk statistic) or a two-sided Wilcoxon rank-sum test if they were not. The categorical variables were expressed as numbers and percentages and were analyzed using contingency table analysis and the chi-squared or Fisher’s test as appropriate. Multivariate analysis was performed to evaluate the impact of risk factors for recurrent RTIs in the study population. All analyses were two-tailed, and *p*-values of <0.05 were considered significant.

## 3. Results

A total of 200 children per group were enrolled. Their demographic and clinical characteristics are reported in Table 1. The groups are comparable, as no statistically significant differences among groups were detected in terms of sex, age, ethnic group, residence, parents’ education, passive smoking from parents, number of siblings, day care attendance, and atopy.

Table 2 shows the impact of OM-85 therapy. In the group of children treated with OM-85, the number of patients who did not experience any new episode of a RTI was significantly lower than that in the group without OM-85. Whereas new RTIs were diagnosed in approximately two-thirds of untreated children, these diagnoses occurred in only approximately one-third of children receiving OM-85 with a reduction of approximately 50% of the risk of new RTI episodes. Similar results were obtained when the total number of RTIs, the number of upper and lower RTIs, and the number of cases with wheezing were considered. Finally, the impact of OM-85 treatment was also significant in reducing the number of children requiring medical visits, antibiotic therapy, and the number of prescribed antibiotic courses. The results were similar in the first and second year of OM-85 administration. None of the study children was hospitalized. Age, gender, and the prevalence of atopy did not influence final results.

Table 3 shows the number of patients reporting ≥3 upper RTIs per year, ≥3 wheezing episodes per year, and ≥3 antibiotic courses per year during the study period, demonstrating the impact of OM-85 in children with the highest tendency to suffer from recurrent RTIs. As shown, the number of patients with the highest tendency to recur and to be treated with antibiotics was significantly lower in the group of patients who had received OM-85 compared with the control group. Additionally, in this case, the effect of OM-85 remained substantially unmodified in the two consecutive years.

Among children treated with OM-85, 11 (5.5%) reported adverse events during the first year of treatment (5 diarrhea, 3 vomit, 2 pyrexia, 1 fatigue) and 9 (4.5%) during the second year (4 diarrhea, 2 vomit, 2 headache, 1 fatigue). All of them were mild and disappeared spontaneously in a few days without requiring treatment withdrawal. Moreover, no increased incidence of infections in locations other than the airways was evidenced in OM-85 children compared with the control group

Among the 200 children treated with OM-85, during the two seasons 49 (24.5%) received the influenza vaccination within one month from the beginning of the first cycle of OM-85. Among these children, 3 (6.1%) reported mild local adverse events (i.e., tenderness in the site of injection), and 2 reported mild systemic adverse events (i.e., fever (max temperature, 38.3 °C) lasting 48 h in one case and inappetence in the other case).

## 4. Discussion

The results of this study showed that OM-85 administered before the winter season was effective in reducing the risk of RTIs in otherwise healthy children with a history of recurrent RTIs. Both the number of children with new episodes and the number of RTIs from which enrolled patients suffered during the study period were significantly lower in children receiving the lysate compared with matched controls. Interestingly, a reduction was demonstrated for both upper RTIs and lower RTIs with wheezing. This last finding seems very clinically important since viral infections in the first years of age are strictly related to the subsequent development of asthma. In contrast, treatments that inhibit inflammation or have antimicrobial activity reduce the structural alterations that follow some viral infections and the development of bronchial hyperresponsiveness [12]. OM-85 seems able to play a relevant role in this regard. It favors correction of the T helper cell (Th) Th1/Th2 imbalance, which is characteristic of the first period of life, through the activation of T regulatory cells, thus reducing the atopic response and tendency to develop asthma [13]. Moreover, under OM-85 treatment, antimicrobial peptides are released, and macrophages are activated with increased secretion of proinflammatory cytokines. Finally, B cell-cytokines are produced, leading to an increased production of polyclonal antiviral and antibacterial immunoglobulins [6].

Regarding prevention of infection-induced wheezing, the data collected from this study confirms what reported by Razi et al. [14]. These authors concluded that children with previous recurrent wheezing experienced a significantly lower number of wheezing attacks when treated with OM-85. Moreover, each attack was two days shorter compared with episodes diagnosed in the placebo controlled group. The reduction in wheezing attacks was 37.9%, which is similar to the one reported in this study.

Moreover, this study suggests previously unknown conclusions. First, it demonstrates that OM-85 can be effective in a number of patients, not only in the first year of use, but also when a second course is administered the following year. Considering that the period during which children remain at risk of recurrent RTIs for immune system immaturity includes several years, this finding is reassuring and can be important from the point of view of the patient, a family, and society. Data collected in this study indicated that even in the second year of administration OM-85 effectiveness is maintained and clinically relevant. A reduction of approximately 50% of RTIs with a consensual reduction of outpatient medical visits and antibiotic prescriptions was evidenced. The positive impact on the quality of life of the patients and their families, and the advantages for the health system are obvious. From an economic point of view, approximately 15 years ago in France, it was calculated that prevention of recurrent acute rhinopharyngitis in at-risk children using OM-85 saved between 6.28 and 303.64 Euro in direct costs for each individual treated preventively [15]. It is highly likely that today this saving would be even greater given the generalized increase in the cost of medical assistance. Interestingly, it was evidenced that some children who did not experience new RTI episodes after the first OM-85 administration maintained this favorable response even after the second OM-85 course. Whether this finding is related to external unknown factors, or indicates that the bacterial lysate favors a more rapid immune system maturation is unknown. However, the problem deserves attention, and further studies to clarify this aspect are needed.

Furthermore, OM-85 was safe and well tolerated. No severe adverse events were reported during both OM-85 courses, confirming the results of previously performed clinical trials, and highlighting that even repeated OM-85 courses did not cause severe clinical problems. Finally, despite the limited number of patients, our study confirmed the safety of the concomitant administration of OM-85 and influenza vaccine. This last finding confirms previous data [16] and appears important because OM-85 and the influenza vaccine are both recommended in children with recurrent RTIs to reduce respiratory recurrences and seem to act with a synergic effect.

This study had some limitations. It was a retrospective study and not a randomized controlled trial that would have made possible to confirm OM-85 efficacy with a higher level of evidence. The study sample was relatively limited. No immunologic study was performed and no biochemical data pre- and post-OM-85 administration were available. Finally, although it is highly likely that most of them were of viral origin, etiology of infections from which enrolled children suffered was not evaluated. 

## 5. Conclusions

This study suggests that OM-85 can effectively and safely reduce the risk of new episodes in children with recurrent RTIs regardless the etiology and that a second course of lysate administration can be useful to maintain protection, particularly when the diagnosis of recurrent RTIs is made in younger children for whom it is likely that definitive maturation of the immune system still requires a long time.

## Figures and Tables

**Table 1:** Baseline characteristics of children treated or not treated with OM-85.

Characteristic	Treatment with OM-85 for 2 Consecutive Seasons (*n* = 200)		Not Treated with OM-85 (*n* = 200)	
	*n*	%	*n*	%
Sex				
Male	116	58.0	109	54.5
Female	84	42.0	91	45.5
Age at enrollment (years)				
1–2	52	26.0	46	23.0
3–4	110	55.0	119	59.5
5–6	38	19.0	35	17.5
Mean ± SD	3.7 ± 1.3		3.9 ± 1.7	
Ethnic group				
Caucasian	170	85.0	163	81.5
Any other	30	15.0	22	18.5
Residence				
Urban area	118	59.0	110	55.0
Rural area	82	41.0	90	45.0
Education (parents)				
Primary/secondary	11	5.5	9	4.5
High school degree (at least 1 parent)	112	56.0	121	60.5
University degree (at least 1 parent)	77	38.5	70	35.0
Passive smoking (from parents)				
No	127	63.5	131	65.5
Yes (at least 1 parent)	73	36.5	69	34.5
No. of siblings				
0	79	39.5	31	15.5
1	101	50.5	113	56.5
2+	20	10.0	56	28.0
Attending nursery/primary school				
No	27	13.5	34	17.0
Yes	173	86.5	166	83.0
Atopy				
No	131	65.5	139	69.5
Yes	69	34.5	61	30.5

No statistically significant differences were detected between groups.

**Table 2:** Clinical and socioeconomic impact of OM-85 during follow-up, according to treatment group.

	First Year		Second Year	
	Treated with OM-85 (*n* = 200)	Untreated with OM-85 (*n* = 200)	Treated with OM-85 (*n* = 200)	Untreated with OM-85 (*n* = 200)
Children with respiratory infections (any)	*n* (%)	*n* (%)	*n* (%)	
No	128 (64.0) *	72 (36.0)	134 (67.0) ^	79 (39.5)
Yes	72 (36.0)	128 (64.0)	66 (33.0)	121 (60.5)
Children with wheezing (any)				
No	167 (83.5) *	140 (70.0)	179 (89.5) ^	152 (76.0)
Yes	33 (16.5)	60 (30.0)	21 (10.5)	48 (24.0)
Children treated with antibiotics (any)				
No	139 (69.5) *	90 (45.0)	146 (73.0) ^	99 (49.5)
Yes	61 (30.5)	110 (55.0)	54 (27.0)	101 (50.1)
Children who required an outpatient medical visit				
No	91 (45.5) *	51 (25.5)	103 (51.5) ^	62 (31.0)
Yes	109 (54.5)	149 (74.5)	97 (48.5)	138 (69.0)
Number of infections				
URTI, Mean ± SD	0.41 ± 0.39 *	0.76 ± 0.49	0.36 ± 0.25 ^	0.61 ± 0.46
Wheezing, Mean ± SD	0.22 ± 0.16 *	0.36 ± 0.31	0.16 ± 0.10 ^	0.31 ± 0.27
LRTI, Mean ± SD	0.16 ± 0.33	0.22 ± 0.58	0.06 ± 0.16 ^	0.14 ± 0.19
Socioeconomic impact				
Antibiotic courses, Mean ± SD	0.37 ± 0.61 *	0.64 ± 0.58	0.31 ± 0.44 ^	0.55 ± 0.48
Outpatient medical visits, Mean ± SD	0.36 ± 0.68 *	0.64 ± 0.58	0.28 ± 0.46 ^	0.55 ± 0.44

* *p* < 0.05 and ^ *p* < 0.05 vs. no therapy. No difference in results when comparing the first and second year of OM-85 therapy. URTI: upper respiratory tract infection; LRTI: lower respiratory tract infection.

**Table 3:** Patients reporting ≥3 upper respiratory tract infections (URTIs) per year, ≥3 wheezing episodes per year, and ≥3 antibiotic courses per year during the study period.

	First Year of OM-85	OR (95% CI)	Second Year of OM-85	OR (95% CU)
URTI				
OM-85	43 (21.5%) *	−0.49	36 (18.0%) ^	−0.44
No therapy	92 (46.0%)	(−0.19–0.69)	82 (41.0%)	(−0.13–0.63)
Wheezing				
OM-85	18 (9.0%) *	−0.36	11 (5.5%) ^	−0.27
No therapy	50 (25.0%)	(−0.16–0.61)	43 (21.5%)	(-0.10–0.44)
Antibiotic courses				
OM-85	33 (16.5%) *	−0.47	28 (14.0%) ^	−0.43
No therapy	73 (36.5%)	(−0.16–0.66)	66 (33.0%)	(−0.13–0.73)

CI, confidence interval; OR, odds ratio; * *p* < 0.05 and ^ *p* < 0.05 vs. no therapy.
